# Biocontrol Potential of Rhizobacteria Against *Passalora fulva* and *Tuta absoluta*: A Sustainable Approach for Tomato Protection

**DOI:** 10.3390/plants14172672

**Published:** 2025-08-27

**Authors:** Said Bahoch, Abdessamad Elaasri, Salahddine Chafiki, Fouad Elame, Ahmed Wifaya, El hassan Mayad, Rachid Bouharroud, Redouan Qessaoui

**Affiliations:** 1 Regional Center of Agricultural Research of Agadir, National Institute of Agricultural Research (INRA), Avenue Ennasr, BP415 Rabat Principal, Rabat 10090, Morocco; s.saidbahoch@gmail.com (S.B.); abdsamadelaasri@gmail.com (A.E.); salahddine.chafiki@um6p.ma (S.C.); fouad.elame@inra.ma (F.E.); ahmed.wifaya@inra.ma (A.W.); rachid.bouharroud@inra.ma (R.B.); 2 Laboratory of Biotechnology and Valorization of Natural Resources, Faculty of Science, Ibn Zohr University of Agadir, Agadir 80000, Morocco; 3 AgroBioSciences Department (AgBS), Mohammed VI Polytechnic University (UM6P), Ben Guerir 43150, Morocco

**Keywords:** tomato, plant growth-promoting rhizobacteria, *P. fulva*, *T. absoluta*, biocontrol, sustainable agriculture

## Abstract

Plant growth-promoting rhizobacteria (PGPR) offer a sustainable strategy for enhancing crop productivity and suppressing phytopathogens. In this study, seven bacterial isolates obtained from the rhizosphere of healthy tomato plants were evaluated for their antagonistic activity against the fungal pathogen *Passalora fulva*, the leaf miner *Tuta absoluta*, and their effects on tomato growth. In vitro dual-culture assays revealed that isolates IQR1, IQR2, IQR3, and IQR5 significantly inhibited *P. fulva* mycelial growth, with inhibition rates exceeding 35%. Volatile organic compounds (VOCs) produced by the bacterial isolates exhibited considerable antifungal activity, with IQR5, IQR1, and IQR2 achieving over 84% inhibition. Molecular identification based on 16S rDNA sequencing indicated that these isolates belong to distinct taxa: *Leucobacter aridicolis* (ON799334.1) (genus *Leucobacter*), *Paenochrobactrum* sp. (JF804769.1) (genus *Paenochrobactrum*), an uncultured bacterium (JQ337400.1) (genus *Psychrobacter*), and marine bacterium AK6_052 (KF816539.1) (genus *Brevundimonas*). Under greenhouse conditions, isolates IQR3, IQR5, and IQR1 reduced disease incidence of *P. fulva* to 20–26%. The same isolates also promoted plant growth, enhancing stem height and collar diameter. In addition, IQR5 significantly reduced *T. absoluta* larval density and foliar damage, with the number of larvae per leaflet decreasing to 1.42, compared to 3.20 in the control. These findings highlight the potentials of these rhizobacterial strains—particularly IQR5—as effective biocontrol agents and biofertilizers for integrated pest and disease management in tomato cultivation.

## 1. Introduction

Tomato (*Solanum lycopersicum* L.) is one of the most important horticultural crops cultivated worldwide, due to its economic and nutritional value [1]. In Morocco, the crop holds significant commercial importance, particularly in the Souss-Massa region, where greenhouse cultivation spans approximately 16,000 hectares, yielding an estimated 1.5 million tons annually [2]. However, tomato production is frequently threatened by a wide range of biotic and abiotic stresses, with fungal diseases and insect pests being among the most detrimental.

Among the fungal pathogens affecting tomato, *Cladosporium fulvum* (syn. *Passalora fulva*) is a major causal agent of leaf mold disease, inflicting considerable economic losses in protected cultivation systems [3]. This pathogen primarily infects leaves, stems, seedlings, and fruits, particularly under the warm and humid conditions typical of greenhouses [4]. Simultaneously, tomato crops face substantial pressure from insect pests such as the tomato leaf miner, *Tuta absoluta* (Meyrick), a highly invasive species belonging to the Gelechiidae family. Its rapid spread and destructive feeding behavior have made it one of the most formidable pests in tomato production globally [5,6].

To mitigate losses caused by these pathogens and pests, tomato growers have traditionally relied on chemical pesticides. For example, Azoxystrobin is a broad-spectrum systemic fungicide that acts as a Qo inhibitor (QoI). It binds to the cytochrome bc1 enzyme complex (Complex III) at the quinol outer (Qo) site. This action disrupts the mitochondrial respiration of plant pathogens, effectively inhibiting their growth and proliferation [7,8]. However, the overuse and misuse of such chemicals have resulted in adverse environmental effects, the development of pesticide resistance, and growing concerns regarding food safety and human health [6,9]. Consequently, the demand for sustainable and eco-friendly pest management strategies has increased, highlighting the need for effective biological alternatives.

Biological control, particularly the use of beneficial microorganisms such as plant growth-promoting rhizobacteria (PGPR), has emerged as a promising approach to combat phytopathogens while enhancing plant health [10]. PGPR are naturally occurring soil bacteria that can colonize plant roots and promote plant growth directly, through nutrient acquisition or hormone production, and indirectly, by suppressing pathogens through various antagonistic mechanisms [11,12,13]. Species from genera such as *Bacillus*, *Pseudomonas*, and actinobacteria have demonstrated significant biocontrol potential against a variety of plant pathogens [11,14,15,16,17,18].

Multiple mechanisms underlie the biocontrol activity of PGPR, including the secretion of antibiotic compounds, competition for nutrients and space, siderophore-mediated iron sequestration, the production of extracellular lytic enzymes, and the induction of systemic resistance in the host plant [19,20,21,22]. The elicitation of induced systemic resistance (ISR) enhances the plant’s defense responses without directly targeting the pathogens. Several studies reported that endophytic bacteria have the capacity to colonize internal plant tissues, providing systemic protection against a wide range of pathogens. This protective effect is largely mediated through the activation of Induced Systemic Resistance (ISR), a defense mechanism triggered by microbe-associated molecular patterns (MAMPs) that are recognized by specific plant receptors. This recognition initiates signaling cascades involving the jasmonic acid and ethylene pathways, thereby priming the plant’s immune system for a faster and stronger response upon pathogen attack. Consequently, plants exhibit a reduction in disease severity and an enhancement in overall health, thereby demonstrating an improvement in their resilience to diverse biotic stresses [23,24].

In addition to their biocontrol potential, plant growth-promoting rhizobacteria (PGPR) enhance plant development through multiple mechanisms, including phosphate solubilization, siderophore production, nitrogen fixation, and phytohormone synthesis [21,25,26]. These functions improve seed germination, plant vigor, and growth by increasing nutrient availability. Sultana et al. (2021) [27] demonstrated that PGPR-derived metabolites, such as siderophores and plant hormones—including indole-3-acetic acid (IAA) [28,29], gibberellic acid [30], and auxin [22,31] play crucial roles in stimulating plant growth. Additionally, Mishra et al. (2010) [32] reported that Pseudomonas fluorescens MA-4 efficiently produced ammonia and significantly increased geranium biomass. These characteristics establish PGPR as a valuable biofertilizer for promoting sustainable agriculture [22].

Given the dual challenge of fungal diseases and insect pests in tomato production, the present study was designed to explore the potential of rhizospheric bacterial isolates as biocontrol agents. Specifically, we investigated the in vitro and in vivo efficacy of selected PGPR strains in suppressing *P. fulva*, reducing *T. absoluta* infestation, and promoting the vegetative growth of tomato plants. This integrative approach aims to identify sustainable microbial candidates for integrated pest and disease management strategies in tomato cultivation.

## 2. Results

### 2.1. Isolation of Rhizobacteria from Tomato Rhizosphere

A total of seven bacterial isolates, designated IQR1 through IQR7, were successfully isolated from the rhizosphere of tomato plants cultivated in a greenhouse at the National Institute for Agronomic Research (INRA) experimental station. These isolates were selected based on their distinct morphological characteristics (color and form) on nutrient agar and were further evaluated for their antagonistic potential against *P. fulva*.

### 2.2. In Vitro Antagonism of P. fulva by Bacterial Isolates

**Figure 1:**
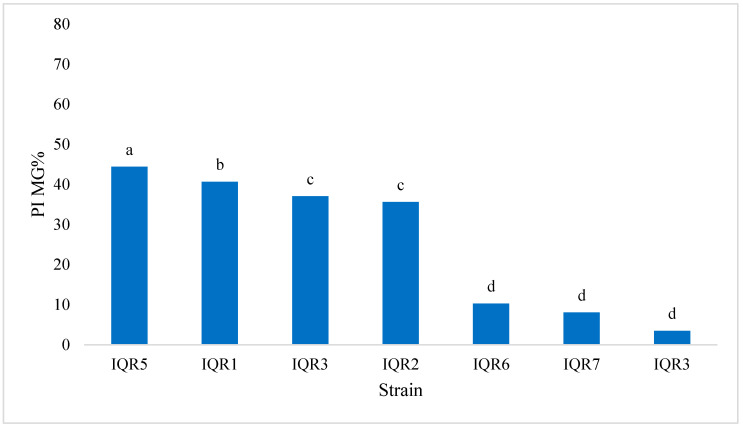
Effects of seven bacterial isolates on *P. fulva* mycelial growth. Bars with the same letters are not significantly different according to the Newman–Keuls test (*p* < 0.05). PIMG: percentage inhibition of mycelial growth, %.

**Figure 2:**
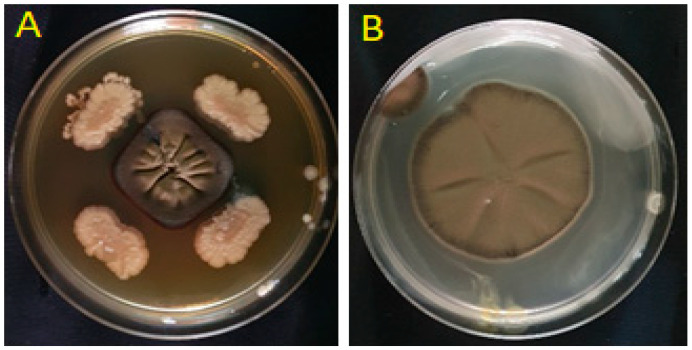
Inhibition of *P. fulva* mycelial growth in PDA medium: (**A**) treated with isolate IQR1 and (**B**) control.

Among the seven tested isolates, four (IQR1, IQR2, IQR3, and IQR5) exhibited significant inhibitory activity against *P. fulva* in the dual-culture assay. These isolates exhibited inhibitions of 41.00% (IQR1), 35.60% (IQR2), 37.08% (IQR3), and 44.44% (IQR5), respectively (Figure 1). This antagonistic activity was visually confirmed by the observation of reduced mycelial growth in comparison to the control group (Figure 2).

### 2.3. Molecular Identification and Phylogenetic Characterization

**Figure 3:**
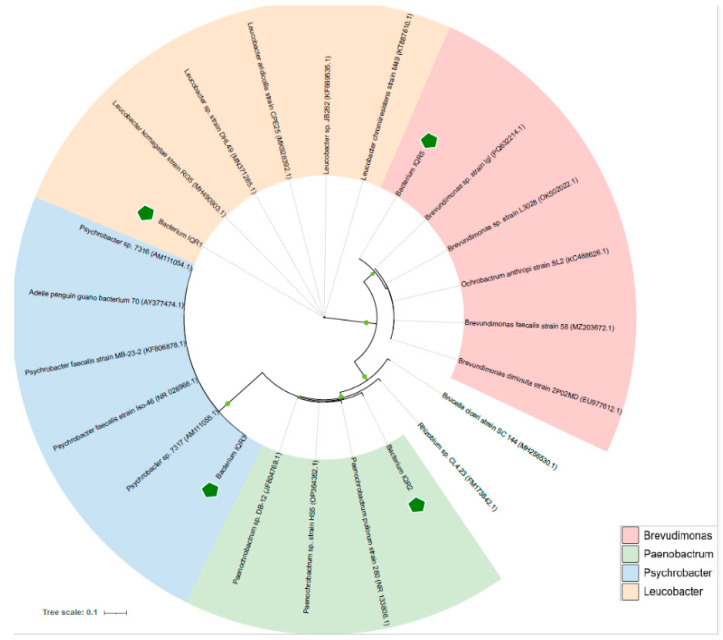
Phylogenetic tree produced by UPGMA analysis with Bionumerics software v.7.6 showing the relationships between four strains and other related bacterial pathovars based on a partial 16s RNA sequence.

Molecular identification based on 16S rDNA sequencing revealed that the four antagonistic isolates belong to distinct bacterial genera. Isolate IQR1 (accession number ON799334.1) showed 99.18% sequence similarity with *Leucobacter aridicolis*, while IQR2 (JF804769.1) shared 96.25% similarity with *Paenochrobactrum* sp. Isolates IQR3 (JQ337400.1) and IQR5 (KF816539.1) displayed 100% identity with an uncultured bacterium and marine bacterium AK6_052, respectively. Phylogenetic analysis using UPGMA confirmed that the isolates belong to the genera *Leucobacter*, *Paenochrobactrum*, *Psychrobacter*, and *Brevundimonas* (Table 1 and Figure 3).

### 2.4. Volatile Organic Compound (VOC) Activity Against P. fulva

**Figure 4:**
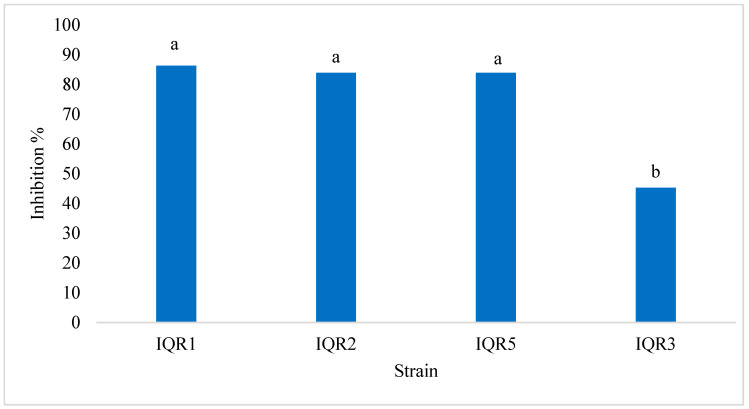
Effects of volatile compounds on *P. fulva* inhibition. Bars with the same letters are not significantly different according to the Newman–Keuls test (*p* < 0.05).

**Figure 5:**
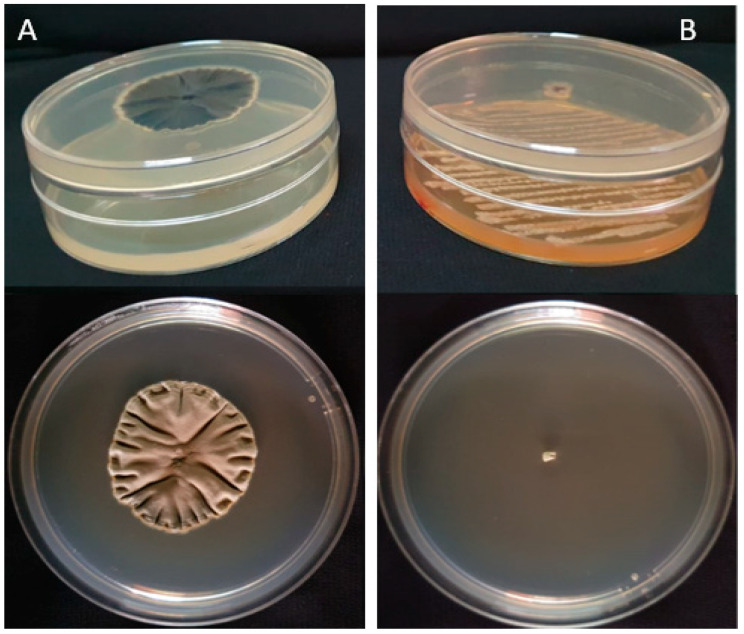
Antagonistic effect of volatile antifungal compounds on growth of *P. fulva*: (**A**) control and (**B**) treated with IQR5 strain.

The four selected isolates also demonstrated significant antifungal activity via volatile organic compounds. The percentage inhibition of *P. fulva* mycelial growth (PIVOC) ranged from 45% to 86%. The highest inhibition was recorded for isolates IQR5 (86%), IQR1 (84%), and IQR2 (84%) (Figure 4). This was visually confirmed by substantial growth suppression of the pathogen on VOC-exposed plates (Figure 5).

### 2.5. Hydrogen Cyanide Production

**Figure 6:**
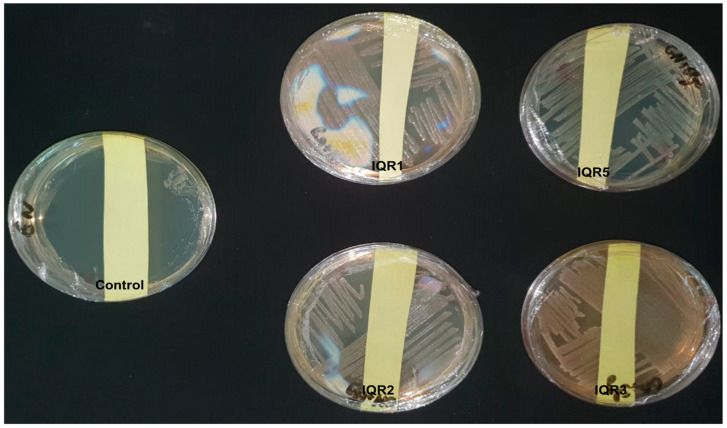
Indication of hydrogen cyanide production by four isolates.

None of the four isolates produced hydrogen cyanide, as evidenced by the lack of color change in the picric acid-impregnated filter paper (Figure 6). This indicates that their antagonistic effects are not mediated by hydrogen cyanide production.

### 2.6. Promotion of Tomato Growth Under Greenhouse Conditions

**Figure 7:**
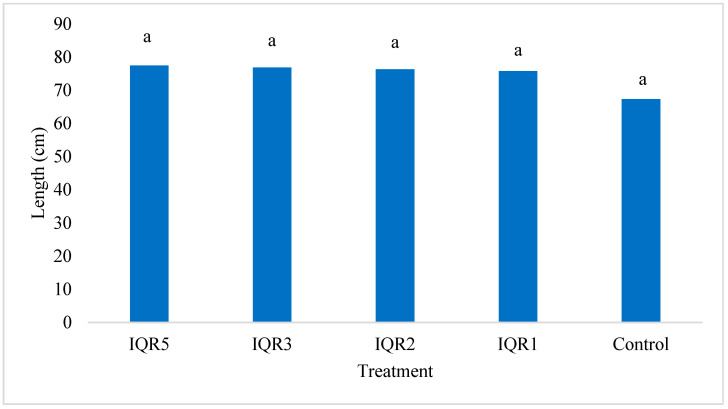
Effects of four bacterial isolates on tomato plant height. Bars with the same letters are not significantly different according to the Newman–Keuls test (*p* < 0.05).

**Figure 8:**
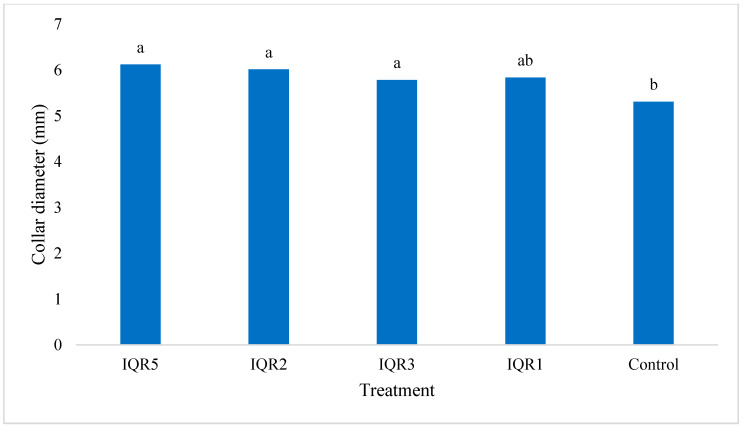
Effects of four bacterial isolates on the stem diameter of tomato plants. Bars with the same letters are not significantly different according to the Newman–Keuls test (*p* < 0.05).

The four isolates were evaluated for their effects on tomato growth parameters under greenhouse conditions. All four isolates significantly increased plant height compared to the control plants (67.0 cm). Although the highest numerical value was observed with isolate IQR5 (77.0 cm), statistical analysis revealed no significant differences among the four isolate treatments (Figure 7). In terms of stem diameter, all isolates significantly enhanced stem diameter, with the maximum values recorded for IQR3 (5.79 mm) and IQR5 (6.12 mm), compared to 5.31 mm in the control (Figure 8).

**Figure 9:**
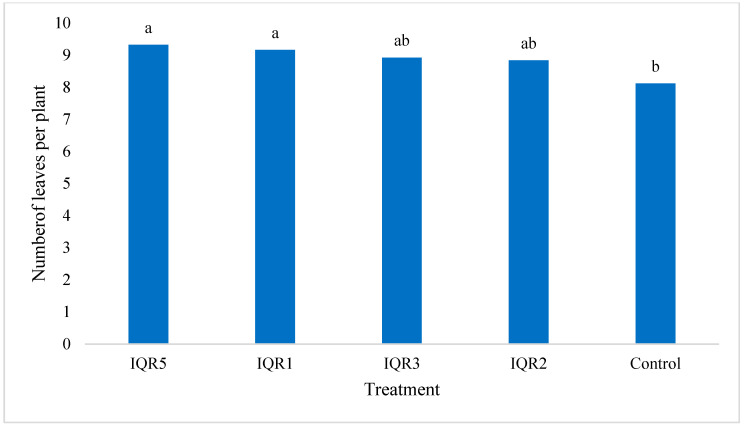
Effects of four bacterial isolates on the number of leaves per tomato plant. Bars with the same letters are not significantly different according to the Newman–Keuls test (*p* < 0.05).

The number of leaves was also influenced by bacterial inoculation. Isolates IQR2 and IQR5 significantly increased the average number of leaves per plant to nine, compared to eight in the control (Figure 9).

### 2.7. Biocontrol Activity Against P. fulva Under Greenhouse Conditions

**Figure 10:**
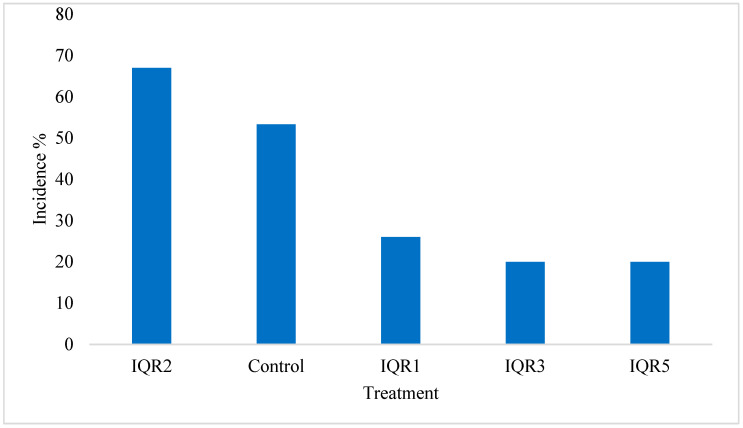
Effects of bacterial isolates on *P. fulva* incidence on tomato plants under greenhouse conditions.

Greenhouse trials confirmed the biocontrol potential of the isolates against *P. fulva*. Disease incidence (DI) was significantly reduced in plants treated with IQR3 and IQR5 (20%), followed by IQR1 (26%). Conversely, IQR2 (53%) and the control treatment (67%) showed the highest rates of disease incidence (Figure 10).

### 2.8. Effects of Bacterial Isolates on Tuta absoluta Infestation

**Figure 11:**
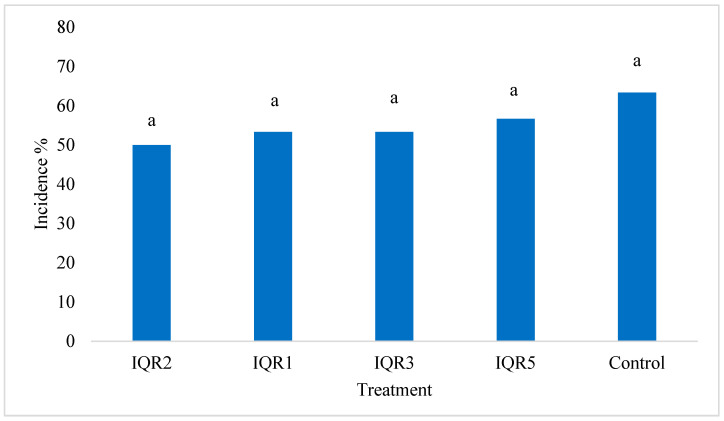
Effects of four isolates on *T. absoluta* incidence. Bars with the same letters are not significantly different according to the Newman–Keuls test (*p* < 0.05).

Assessment of *T. absoluta* infestation showed no statistically significant differences in overall plant-level incidence among treatments (Figure 11). However, the treatments showed a clear numerical trend toward reduced infestation, with percentages of 50% (IQR2), 53% (IQR1), and 53% (IQR3) relative to the 63% infestation rate of the control.

**Figure 12:**
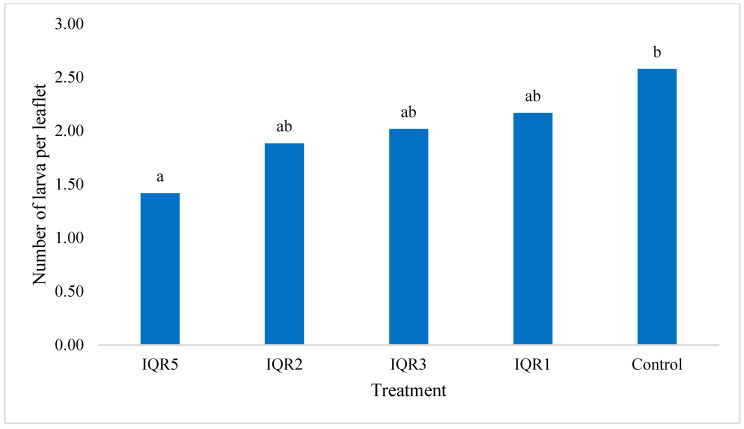
Effects of four isolates on the number of larvae per leaflet. Bars with the same letters are not significantly different according to the Newman–Keuls test (*p* < 0.05).

A significant reduction in the number of larvae per leaflet was recorded in all treatments compared to the control. Isolate IQR5 was the most effective, with an average of 1.42 larvae per leaflet, followed by IQR2 (1.88), IQR3 (2.02), and IQR1 (2.17) (Figure 12). Leaf damage was also significantly reduced by some treatments.

**Figure 13:**
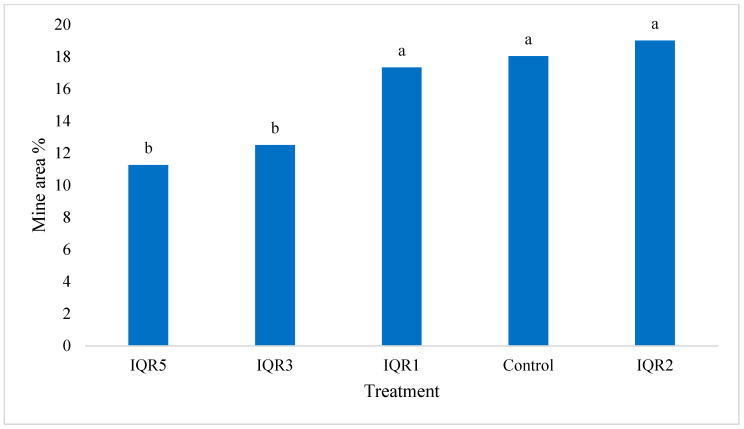
Effects of four isolates on the mined area in infected leaves. Bars with the same letters are not significantly different according to the Newman–Keuls test (*p* < 0.05).

Isolates IQR5 and IQR3 achieved the greatest reductions in mined leaf area, with values of 11.25% and 12.50%, respectively, compared to 18.03% in the control (Figure 13). These results suggest that IQR5 and IQR3 are the most promising candidates for reducing the damaging effects of *T. absoluta* larvae.

### 2.9. Principal Component Analysis (PCA)

**Figure 14:**
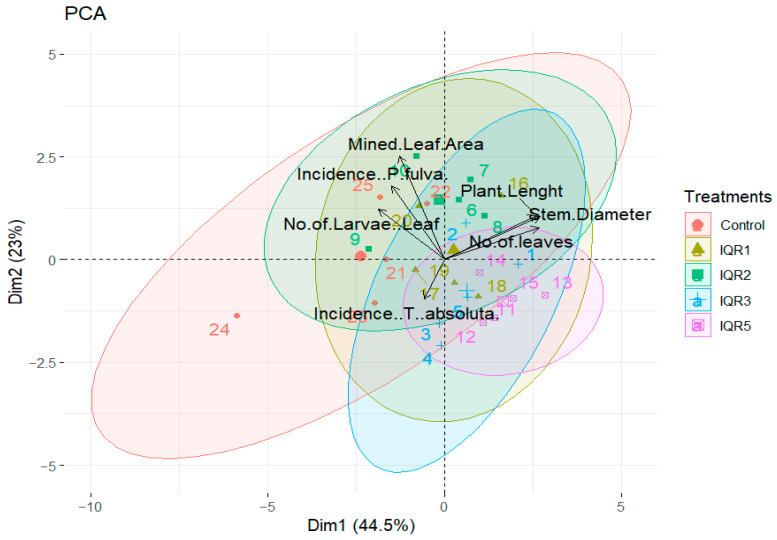
Principal component analysis (PCA) biplot showing correlations among agronomic parameters and *T. absoluta* infestation variables in tomato plants treated with rhizobacterial strains.

PCA revealed a clear separation of treatments based on plant growth and pest infestation parameters (Figure 14). The first principal component (Dim1), which accounts for 44.5% of the total variance, distinctly separates growth traits (plant length, stem diameter, number of leaves) from pest metrics (incidence of *T. absoluta*, number of larvae per leaf, mined leaf area). Growth parameters cluster on the positive side of Dim1, while pest indicators are grouped on the negative side, indicating a strong inverse relationship. This highlights the potential effects of those isolates in enhancing tomato plant growth and health.

## 3. Discussion

The rhizosphere of healthy tomato plants was shown to harbor a diverse and functionally active microbial community, including bacterial taxa with promising biocontrol and plant growth-promoting capabilities. In this study, four bacterial isolates—identified as *Leucobacter aridicolis* (IQR1), *Paenochrobactrum* sp. (IQR2), an uncultured bacterium (IQR3), and marine bacterium AK6_052 (IQR5)—exhibited distinct phylogenetic profiles and strong biocontrol potential. These genera (*Leucobacter*, *Paenochrobactrum*, *Psychrobacter*, and *Brevundimonas*) have previously been associated with beneficial traits in agroecosystems. For example, *Leucobacter* species have been implicated in enhancing drought tolerance in rice [33], while *Paenochrobactrum* has been noted for its attractive properties on dipteran adults [34]. Additionally, psychrotolerant genera, such as *Psychrobacter* and *Brevundimonas*, have been increasingly recognized for their contributions to plant growth and biocontrol [35,36,37].

The in vitro assays revealed that all four selected isolates significantly inhibited *P. fulva*, with IQR5 (*Marine bacterium* AK6_052) and IQR1 (*Leucobacter aridicolis*) demonstrating the strongest antagonistic effects. These findings are consistent with earlier reports highlighting the effectiveness of bacterial antagonists in suppressing leaf mold pathogens. For instance, *Bacillus subtilis* strain SJ-2 inhibited *P. fulva* growth by more than 80% [38], while *B. subtilis* strain WXCDD105 achieved 94.4% inhibition [39]. Similarly, *Bacillus velezensis* NKG-2 exhibited 50.7% inhibition against *P. fulva* [40]. The mode of action in these studies is often linked to the production of antifungal metabolites and hydrolytic enzymes that compromise fungal cell integrity [41,42,43,44,45].

In our study, none of the four isolates produced hydrogen cyanide (HCN), suggesting that their biocontrol activities are mediated through other mechanisms. Volatile organic compounds (VOCs) emitted by these isolates significantly inhibited *P. fulva*, with IQR5 and IQR1 achieving inhibition levels exceeding 84%. VOCs are well-documented for their antifungal properties and have been shown to interfere with spore germination and mycelial growth [45]. Therefore, the observed antifungal effect is likely due to these bioactive volatiles, rather than cyanogenic activity.

Under greenhouse conditions, the isolates significantly reduced disease incidence caused by *P. fulva*, with IQR3 and IQR5 lowering disease incidence to 20%, and IQR1 to 26%. These results are in line with previous studies demonstrating the effectiveness of *Bacillus* strains in suppressing leaf mold under semi-controlled conditions [39,46]. The observed biocontrol activity in planta may also be associated with the induction of systemic resistance (ISR), a process by which PGPR prime the plant immune system for enhanced defense responses against pathogens [19,20].

The bacterial isolates demonstrated a clear effect against *Tuta absoluta*, with a notable reduction in pest damage. Isolate IQR5 exhibited the highest larval mortality and lowest mined leaf area, while isolates IQR3 and IQR2 also showed significant reductions. Although overall plant-level infestation incidence was not statistically different from the control, the isolates significantly reduced both the number of larvae per leaflet and the extent of foliar damage. This insecticidal action could result from a direct effect of the bacteria, through the production of insecticidal compounds—such as enzymes—that can kill insects or disrupt their behavior, or, alternatively, from an indirect effect via the induction of systemic resistance in tomato plants. Similar biocontrol effects have been documented for PGPR producing chitinases and biosurfactants—compounds that disrupt the cuticles and digestive tracts of insect larvae [47,48]. *Bacillus* spp., such as *B. subtilis*, *B. amyloliquefaciens*, and *B. thuringiensis*, have been widely reported to exert larvicidal activity against *T. absoluta* via the production of Cry and Cyt δ-endotoxins [49,50,51].

Although none of the isolates in our study were *Bacillus* spp., their biocontrol performance suggests that alternative bacterial genera may also possess insecticidal traits, possibly via similar or complementary mechanisms.

This study additionally confirmed the effect of the tested strains on tomato plant growth, demonstrating their significant growth-promoting effects under greenhouse conditions compared with the control. The bacterial treatments significantly increased stem diameter and the number of leaves per plant, indicating their ability to enhance vegetative development. These effects can be attributed to the strains’ capacity to improve nutrient availability to plants, as well as their production of phytohormones such as auxins and cytokines, which are known to stimulate plant growth. In a similar context, Qessaoui et al. (2019) [22] reported that *Pseudomonas* spp. strains Q6B, Q14B, Q7B, Q1B, and Q13B significantly promoted plant length, collar diameter, and leaf number in tomato compared to the control. In a similar study, Gholami et al. (2009) [52] demonstrated that seed inoculation with six bacterial strains—P. putida R-168, P. fluorescens R-93, P. fluorescens DSM 50090, P. putida DSM 291, A. lipoferum DSM 1691, and A. brasilense DSM 1690—significantly enhanced seed germination and seedling vigor in maize.

The PCA results further supported the link between enhanced plant vigor and reduced pest pressure. Positive correlations among growth parameters (plant height, stem diameter, leaf number) and negative associations with *T. absoluta* damage suggest that healthier plants may be more resilient to infestation, or that the bacterial isolates directly suppressed pest development.

Taken together, these findings demonstrate the multifaceted role of rhizobacteria in promoting plant health and controlling key tomato pathogens and pests. IQR5, in particular, consistently outperformed other isolates across in vitro and in vivo assays, making it a strong candidate for development as a microbial inoculant in integrated pest and disease management strategies

## 4. Materials and Methods

### 4.1. Isolation of Rhizosphere Bacteria

Soil samples were collected from the rhizospheres of healthy tomato plants grown at the INRA experimental station in Melk Zhar, Belfaa, Morocco (30°02′42.2″ N, 9°33′13.4″ W). For each replicate, approximately 500 g of soil was sampled from greenhouse-grown tomato roots and stored at 4 °C before analysis. The rhizosphere bacteria were isolated using a modified version of the protocol, described by Dommergues and Mangenot [53]. Briefly, after gentle removal of loosely adhering soil, 1 g of rhizosphere soil was suspended in 9 mL of sterile physiological saline (0.9%) and shaken at 120 rpm for 2 min. Serial dilutions were prepared, and 0.1 mL aliquots from each dilution were plated onto nutrient agar medium and incubated at 28 °C for 48 h. All procedures were performed in triplicate. Distinct colonies were sub-cultured twice for purification, then maintained on yeast dextrose carbonate (YDC) agar at 4 °C and stored at −80 °C in 40% glycerol [54].

### 4.2. Antagonistic Effects

The antagonistic activity of the bacterial isolates against *P. fulva* was evaluated using the dual-culture technique, as described by Patel and Brown [55]. Petri dishes were prepared using a 70:30 mixture of potato dextrose agar (PDA) and glucose–nutrient agar (GN). Four aliquots of each bacterial isolate, taken from 48 h old cultures, were placed 2.5 cm from the center of the plate. After 24 h of incubation at 28 °C, a 5 mm mycelial plug of *P. fulva* was placed at the center of each plate. Control plates contained only the fungal pathogen. All treatments were conducted in triplicate and incubated at 26 °C for 12 days. The degree of inhibition was calculated using the percentage inhibition of mycelial growth (PIMG), according to Whipps [56], as follows:

PIMG (%) = (R1 − R2)/R1 × 100

where R1 is the radial growth of the fungal colony in control, and R2 is the radial growth in the presence of bacterial antagonists.

### 4.3. Molecular Identification of Bacterial Isolates

Isolates demonstrating significant in vitro antagonism were selected for molecular identification. Each isolate was cultured in 5 mL of Lysogeny Broth (LB) and incubated at 30 °C for 18 h with shaking at 150 rpm. Genomic DNA was extracted using the DNeasy Blood and Tissue Kit (Canvax Biotech, Valladolid, Spain), following the manufacturer’s protocol. The 16S rDNA region was amplified by PCR using universal primers FD1 (5′-AGAGTTTGATCATGGCTCAG-3′) and RD1 (5′-AAGGAGGTGATCCAGCCGCA-3′) [57]. PCR products were purified using the ExoSAP-IT™ PCR Product Cleanup Reagent (Affymetrix Inc., Santa Clara, CA, USA). Sequencing was performed unidirectionally using the BigDye™ Terminator v3.1 Cycle Sequencing Kit (ThermoFisher, Waltham, MA, USA) and analyzed using the Applied Biosystems SeqStudio Genetic Analyzer (Applied Biosystems, Waltham, MA, USA). The obtained sequences were submitted to GenBank and used for BLAST searches against the NCBI database. Phylogenetic trees were constructed using UPGMA clustering with Bionumerics v7.6.

### 4.4. Volatile Organic Compound (VOC) Assay

To assess the inhibitory activity of bacterial volatile compounds against *P. fulva*, a sealed plate assay was conducted as described by Fiddaman and Rossall [58]. Each bacterial isolate was cultured on King’s B medium and incubated at 28 °C for 48 h. The lids of these Petri dishes were then replaced with separate PDA plates inoculated with a 6 mm mycelial plug of *P. fulva*. The two plates were sealed together using parafilm, allowing only volatile diffusion. Control setups lacked bacterial cultures. The percentage inhibition of mycelial growth (PIVOC) was calculated after five days of incubation at 25 °C, using the following formula:

$$PIVOC=\frac{r1-r2}{r1}\times 100$$

where r1 and r2 represent radial growth in control and treated plates, respectively [59].

### 4.5. Hydrogen Cyanide Production

The qualitative production of hydrogen cyanide was determined following the method of Bakker and Schippers [60]. Isolates were streaked on King’s B agar supplemented with 4.4 g/L glycine. A Whatman filter paper saturated with 0.5% picric acid, and 2% sodium carbonate was affixed to the lid of each inverted Petri dish. Plates were sealed with parafilm and incubated at 30 °C for 96 h. A color change from yellow to brown indicated hydrogen cyanide production [61].

### 4.6. Greenhouse Evaluation of Antagonistic Activity Against P. fulva

The efficacy of selected isolates against *P. fulva* was tested under greenhouse conditions. Bacterial inocula were incorporated into a peat/sand (2:1) substrate at a concentration of 10^8^ CFU/g. Specifically, 300 mL of bacterial suspension was thoroughly mixed into 30 kg of substrate per isolate. Tomato seedlings (*S. lycopersicum*, cv. Bellatrix) at the two-true-leaf stage were transplanted into treated pots arranged in a randomized complete block design (RCBD) with six replicates per treatment, each comprising five plants. Drip irrigation and an optimized fertilization regime were used to minimize abiotic stress. Evaluated growth parameters included plant height, stem diameter at the collar, and number of leaves.

Thirty days post-transplantation, plants were foliar-sprayed with bacterial suspensions, followed by application of *P. fulva* spore suspensions 15 min later, ensuring that both adaxial and abaxial leaf surfaces were treated [62].

Disease incidence (DI) is calculated as the proportion of diseased plants relative to the total number of plants subjected to the same treatment [63]. In this study, it was determined as the number of plants showing leaf spot symptoms (a), relative to the 30 plants treated with the same isolate (N):

$$DI=\frac{a}{N}\times 100$$

### 4.7. Evaluation of the Effects of Isolates on Tuta absoluta Under Greenhouse Conditions

The effect of the isolates on the tomato leaf miner (*T. absoluta*) was tested on three-month-old plants. After natural *T. absoluta* infestation, each plant was treated again with 30 mL of bacterial solution (10^8^ CFU/mL). Threatened plants were used for each treatment. The effect on *T. absoluta* was assessed 7 days after treatment through the following parameters:

Incidence: The incidence was determined as the number of infested plants relative to the total number of plants (30) treated with the same bacterial isolate. In each experimental unit, the number of infested leaflets per plant was recorded.

Number of larvae per leaflet: A sample of four randomly selected leaflets from the middle stratum of the plants in each experimental unit was collected to count the number of larvae.

Percentage of mined leaf area: A random sample of four leaflets per experimental unit was selected. The percentage of the leaf area mined by *T. absoluta* larvae was visually estimated relative to the total leaflet area. The percentage of leaf damage ranged from 0% to 100%.

### 4.8. Data Analysis

All data were subjected to analysis of variance (ANOVA), and means were compared using the Newman–Keuls test at a significance level of *p* < 0.05. Results are presented as means ± standard deviations. Statistical analyses were performed using Minitab 19 software (Minitab LLC., State College, PA, USA).

## 5. Conclusions

This study provides a foundational understanding of sustainable strategies for tomato protection by demonstrating the multifunctional efficacy of selected plant growth-promoting rhizobacteria (PGPR). Beyond confirming their established biocontrol and growth-promoting properties, our findings highlight the potential of bacterial genera, such as *Leucobacter*, *Paenochrobactrum*, *Psychrobacter*, and *Brevundimonas*, to serve as viable alternatives to conventional agrochemicals by enhancing plant vitality and controlling the impacts of fungal and insect pests. This research supports a strategy of Integrated Pest and Disease Management (IPDM) and underscores the critical need to broaden the search for biological control agents beyond model organisms, particularly given the robust, multi-target efficacy of isolate IQR5, which positions it as a promising candidate for commercial development. To render these findings into practice, future research must prioritize rigorous field trials to validate consistency, a thorough investigation into the molecular mechanisms underlying their bioactivity (secondary metabolites), and the exploration of advanced formulation techniques and synergistic microbial consortia to enhance their reliability and scalability.

## Figures and Tables

**Table 1:** Identification and sequence similarity of bacterial strains based on 16S rRNA gene analysis.

Strain Code	Genus	Accession	Similarity (%)	E-Value	Coverage (%)
IQR1	*Leucobacter*	ON799334.1	99.18	0.0	100
IQR2	*Paenochrobactrum*	JF804769.1	96.25	5 × 10^−170^	95
IQR3	*Psychrobacter*	JQ337400.1	100	0.0	100
IQR5	*Brevundimonas*	KF816539.1	100	8 × 10^−126^	100

## Data Availability

All the data are included in this article.
